# Peripheral Blood Expression of MMPs and DNA Methylation-Related Genes in Women with Recurrent Spontaneous Abortion: A Case-Control Study

**DOI:** 10.12688/f1000research.166306.2

**Published:** 2025-10-06

**Authors:** Maryam Imani, Fateme Zare, Hossein Hadinedoushan, Fateme Rahmani, Farzaneh Fesahat

**Affiliations:** 1 Reproductive Immunology Research Center, Shahid Sadoughi University of Medical Sciences, Yazd, Iran; 2 Department of Immunology, Faculty of Medicine, Shahid Sadoughi University of Medical Sciences, Yazd, Iran

**Keywords:** Recurrent spontaneous abortion; DNA methyltransferases; matrix metallopeptidases; gene expression; peripheral blood.

## Abstract

**Background:**

Recurrent spontaneous abortion (RSA) is one of the most common reproductive complications among women of childbearing age. Several factors—including genetic, anatomical, endocrine, infectious, environmental, and immunological causes—have been implicated in RSA. This study aimed to explore the potential association between RSA and the expression patterns of selected genes involved in critical biological processes related to pregnancy maintenance and miscarriage.

**Methods:**

The relative expression levels of *matrix metallopeptidase 2 (MMP2)*, *matrix metallopeptidase 9 (MMP9)*, *DNA methyltransferase 1 (DNMT1)*, *DNA methyltransferase 3 alpha (DNMT3A)*, and *methylenetetrahydrofolate reductase (MTHFR)* were evaluated using peripheral blood samples from 30 women with a history of two or more spontaneous abortions (cases) and 30 age-matched non-pregnant women with no history of miscarriage and at least one successful pregnancy (controls).

**Results:**

No significant differences in the mRNA expression levels of the target genes were observed between women with RSA and the control group.

**Conclusions:**

Our findings suggest that peripheral blood gene expression profiles of *MMP2, MMP9, DNMT1, DNMT3A*, and *MTHFR* may not adequately reflect tissue-specific changes associated with RSA. Further investigations using relevant tissues, such as placental or decidual samples, are warranted to better understand the molecular mechanisms underlying RSA.

## Introduction

Recurrent spontaneous abortion (RSA), defined as two or more pregnancy losses before 20 weeks of gestation, affects approximately 1–3% of couples of reproductive age. ^1^ Despite advances in reproductive medicine, the etiology of RSA remains unexplained in nearly 50% of cases. ^2^ Among various contributing factors, genetic and epigenetic mechanisms have gained considerable attention due to their essential roles in pregnancy maintenance. Epigenetic markers regulate gene expression to preserve cellular and tissue homeostasis. Disruptions in epigenetic regulation are implicated in numerous conditions, including idiopathic RSA, where specific changes in epigenetic markers are crucial for embryo implantation, tissue remodeling, and the overall success of pregnancy. ^3, 4^ The field of epigenetics offers new insights into the pathogenesis of RSA. Epigenetic modifications—such as DNA/RNA methylation and histone modification—can influence gene expression without altering the DNA sequence and play key roles in cell differentiation, proliferation, and apoptosis. ^5^ Abnormal DNA methylation has been recognized as a potential cause of early pregnancy loss by affecting processes like embryonic development and maternal-fetal interaction. ^6^

Critical reproductive events, including embryonic development, trophoblast invasion, immune tolerance at the maternal-fetal interface, and spiral artery remodeling, are governed by a complex interplay between genetic and epigenetic mechanisms. ^7, 8^ Such dysregulations may contribute to unexplained recurrent pregnancy loss (URPL). Such dysregulations may contribute to unexplained recurrent pregnancy loss (URPL). Understanding these mechanisms could aid in the identification of novel biomarkers and therapeutic strategies for RSA. Recent studies have implicated abnormal DNA methylation in the pathogenesis of RSA, particularly in genes regulating immune tolerance, placental development, and cellular proliferation. Although the exact global prevalence remains uncertain, epigenetic dysregulation is increasingly recognized as a contributing factor in a substantial subset of spontaneous abortion cases. ^9, 10^

DNA methylation—the most widely studied epigenetic modification—is vital for normal embryogenesis and placental function. DNA methyltransferases (DNMTs), including DNMT1 and DNMT3A, are key enzymes that establish and maintain methylation patterns during embryonic and germ cell development. Dysregulated DNA methylation, often due to altered DNMT activity, may impair embryo development and increase the risk of RSA. ^11, 12^

Methylenetetrahydrofolate reductase (MTHFR) is another critical enzyme involved in the methylation cycle, catalyzing reactions that generate methyl groups required for DNA methylation. Polymorphisms in the *MTHFR* gene have been associated with an increased risk of RSA in several studies. ^13^

Matrix metalloproteinases (MMPs) are important in reproductive biology, particularly in implantation and placentation. ^13^ These endopeptidases modulate the extracellular matrix (ECM), facilitating processes such as cell proliferation, migration, and angiogenesis. MMP2 and MMP9, in particular, contribute to ovulation and pregnancy by degrading ECM components to support tissue remodeling. Single nucleotide polymorphisms (SNPs) in the promoter regions of these genes have been reported to influence their expression and activity. ^14, 15^

An imbalance in *MMPs* expression, especially *MMP2* and *MMP9*, may lead to inadequate trophoblast invasion and defective placental formation, which can ultimately contribute to RSA. ^15^ The genes *DNMT1, DNMT3A, MTHFR, MMP2*, and *MMP9* are interconnected through pathways regulating methylation, folate metabolism, and ECM remodeling—each essential for a successful pregnancy.

Despite limited data on the specific contribution of genetic and epigenetic alterations in multiple miscarriages, available evidence supports their involvement in reproductive failure. Therefore, this study aims to assess the expression levels of *MTHFR, DNMT1, DNMT3A, MMP2*, and *MMP9* in the peripheral blood of women with a history of RSA compared to healthy controls. This research seeks to enhance our understanding of RSA pathophysiology and contribute to the development of novel diagnostic and therapeutic approaches.

## Materials and methods

This case-control study was conducted at the Yazd Institute of Reproductive Sciences and involved two distinct groups. The case group comprised 32 women with a history of two or more primary recurrent spontaneous abortions (RSA), with at least three months elapsed since their last miscarriage. The control group included 32 age-matched, non-pregnant women of reproductive age, with no history of miscarriage and at least one successful pregnancy. All participants underwent clinical examination by a gynecologist.

Inclusion criteria for the case group were: absence of male-factor infertility, history of at least two RSAs, and exclusion of other known causes of pregnancy loss, such as genetic, hormonal, anatomical, or chromosomal abnormalities. Written informed consent was obtained from all participants prior to data collection. Peripheral blood samples (5 mL) were collected from each participant, and demographic and reproductive data, including age, number of abortions, and number of pregnancies, were recorded. The study protocol was approved by the Research Ethics Committees of school of public health, Shahid Sadoughi University of Medical Sciences, Yazd, Iran with ethical ID: IR.SSU.SPH.REC.1401.045. All of the participants signed an informed consent before entering the study.

The ethical principles of the Declaration of Helsinki were applied with respect to the confidentiality and veracity of the data collected during the course of the study, which are faithfully presented. Personal identity data and patient privacy were protected. Authorship contributions and transparency in conflicts of interest were reported. ^16^

### RNA extraction and cDNA synthesis

Total RNA was isolated from whole blood using a commercial RNA extraction kit (Yekta Tajhiz Azma, Iran), following the manufacturer’s instructions. RNA concentration and purity were assessed by measuring absorbance at 260 nm and calculating the A260/A280 ratio using a UV spectrophotometer (PhotoBiometer, Eppendorf, Germany). Complementary DNA (cDNA) was synthesized from the extracted RNA using a cDNA synthesis kit (GeneAll, Korea). Synthesized cDNA was stored at −20°C until use.

### Quantitative real-time PCR

Quantitative real-time reverse transcriptase polymerase chain reaction (qRT-PCR) was performed to assess the relative mRNA expression of *MMP2, MMP9, DNMT1, DNMT3A*, and *MTHFR.* *GAPDH* was used as the internal reference gene. Primer sequences were selected based on published studies and validated using Primer-BLAST to ensure specificity. Primer sequences are listed in Table 1.

**Table 1.** Primer sequences used for the real-time polymerase chain reaction.

Genes	Primer sequence (5'->3')	Accession No.	PCR Product (bp)
***MMP2***	**F:**GATACCCCTTTGACGGTAAGGA	NM_001302510.2	112 bp
	**R:**CCTTCTCCCAAGGTCCATAGC		
***MMP9***	**F:**ACGCAGACATCGTCATCCAGT	NM_004994.3	146 bp
	**R:**GGACCACAACTCGTCATCGTC		
***MTHFR***	**F:**TCCCGTCAGCTTCATGTTCT	XM_005263463.5	72 bp
	**R:**TCATACAGCTTTCCCCACCG		
***DNMT1***	**F:**TGGACGACCCTGACCTCAAAT	NM_001379.4	168 bp
	**R:**GCTTACAGTACACACTGAAGCA		
***DNMT3A***	**F:**TATTGATGAGCGCACAAGAGAG	NM_022552.5	111 bp
	**R:**GGGTGTTCCAGGGTAACATTGAG		
***GAPDH***	**F:**AAATCAAGTGGGGCGATGCTG	NM_001256799.3	118 bp
	**R:**GCAGAGATGATGACCCTTTTG		

PCR, Polymerase chain reaction; MMP2, Matrix metallopeptidase 2; MMP9, Matrix metallopeptidase 9; MTHFR, Methylenetetrahydrofolate reductase; DNMT1, DNA methyltransferase 1; DNMT3A, DNA methyltransferase 3 alpha; and GAPDH, Glyceraldehyde-3-phosphate dehydrogenase.

The qRT-PCR reactions were performed using the RealQ Plus 2× Master Mix Green High ROX™ (Ampliqon, Denmark) on a StepOne™ Real-Time PCR System (Applied Biosystems, USA). Each 10 μL reaction mixture contained 1 μL cDNA, 0.5 μL of each forward and reverse primer, 5 μL master mix, and 3 μL nuclease-free water. The thermal cycling conditions included an initial denaturation at 95°C for 10 minutes, followed by 40 cycles of denaturation at 95°C for 15 seconds, annealing at 58°C–64°C (depending on the primer) for 30 seconds, and extension at 72°C for 30 seconds. A melting curve analysis (60°C to 95°C) was performed to confirm the specificity of amplification. Relative gene expression was calculated using the 2 ^−ΔΔCT^ method.

### Statistical analysis

Statistical analysis was performed using SPSS version 16 and GraphPad Prism version 8. Data are presented as mean ± standard error of the mean (SEM). The Kolmogorov-Smirnov test was used to assess data normality. As the data did not follow a normal distribution, the non-parametric Mann–Whitney U test was applied to compare gene expression levels between groups. A p-value of <0.05 was considered statistically significant.

## Results

In this case-control study, 64 participants were enrolled and divided into two groups: cases and controls. There were no significant differences in maternal age between the two groups. The mean ± SD maternal age was 29.1 ± 1.52 years (range: 26–32) in women with a history of recurrent spontaneous abortion (RSA) and 30.03 ± 0.23 years in the control group. The mean ± SD number of RSAs in the case group was 4 ± 1.7. The mean gestational age at the time of the most recent abortion was 11.51 ± 2.15 weeks. In the control group, the mean ± SD number of successful pregnancies was 2.29 ± 0.74). Although the mRNA expression levels of *MMP2, MMP9, DNMT1, DNMT3A*, and *MTHFR* were higher in women with a history of RSA compared to the control group, the differences were not statistically significant ( Table 2).

**Table 2.** Comparison of genes expression levels between the study groups.

Variables	Control	Case	P-Value
***DNMT1***	1.8 ± 0.33	1.94 ±0.45	0.55
***DNMT3A***	1.43 ± 0.21	1.91 ± 0.39	0.87
***MTHFR***	2.34 ± 0.45	2.44 ± 0.55	0.98
***MMP2***	1.40 ± 0.25	1.82 ± 0.29	0.67
***MMP9***	1.13 ± 0.11	1.36 ± 0.21	0.64

Data were presented as mean ± SEM. *P *< 0.05 was regarded as a significant value.

DNMT1: DNA Methyltransferase 1; DNMT3A: DNA methyltransferase 3 alpha; MTHFR: Methylenetetrahydrofolate reductase; MMP2: Matrix metalloproteinases 2; MMP9: Matrix metalloproteinases9.

## Discussion

Recurrent spontaneous abortion (RSA) is a multifactorial condition influenced by genetic, epigenetic, and environmental factors. This study investigated the expression of *DNMT1, DNMT3A, MTHFR, MMP2*, and *MMP9* in the peripheral blood of women with RSA compared to healthy controls. Despite the biological relevance of these genes, no statistically significant differences in their expression were observed between the two groups.

Epigenetic mechanisms, especially DNA methylation, are fundamental for the maintenance of pregnancy, regulating key processes such as embryo implantation, placental development, and maternal-fetal immune tolerance. DNA methyltransferases like DNMT1 and DNMT3A ensure the proper expression of imprinted and immune-related genes, and their dysregulation can impair trophoblast function and immune balance, potentially leading to RSA. Although our data showed a non-significant increase in *DNMT1* and *DNMT3A* expression in the RSA group, this finding does not directly align with previous studies such as those by Li et al., who reported global hypomethylation and reduced *DNMT* expression in chorionic villi and decidua of URPL patients, along with increased TET enzyme activity. The discrepancy may be attributed to differences in tissue type, as our study was conducted on peripheral blood samples, which may reflect systemic or compensatory responses rather than local epigenetic changes at the maternal-fetal interface. ^17^ Additionally, experimental studies have shown that DNMT1 inhibition during early pregnancy reduces DNA methylation and disrupts embryonic attachment, ^18^ emphasizing the importance of balanced methylation for successful implantation. While our findings do not directly reflect local methylation dynamics, they may indicate broader epigenetic alterations relevant to RSA.

It is important to acknowledge that peripheral blood may not accurately reflect gene expression patterns in reproductive tissues such as the decidua or placenta. Our use of peripheral blood was driven by accessibility, but tissue-specific studies are more appropriate for evaluating genes involved in implantation and early placentation.

MTHFR is another key regulator of DNA methylation via folate metabolism. ^19^ Previous studies have reported altered methylation of the *MTHFR* promoter in RSA patients, often associated with reduced enzyme activity and folate imbalance. ^13, 20^ In our study, however, *MTHFR* expression in peripheral blood did not differ significantly between RSA patients and healthy controls. Since we did not assess promoter methylation directly, any inference regarding a disconnect between methylation and gene expression must be interpreted with caution. Moreover, the three-month interval between the last miscarriage and sample collection may have influenced the expression profile, potentially masking transient changes that occur during the acute phase of pregnancy loss. These findings underscore the importance of future studies incorporating both methylation and expression analyses in relevant reproductive tissues and across different time points.

Matrix metalloproteinases (MMP2 and MMP9) are crucial for extracellular matrix remodeling during early placental development. Although our findings showed a non-significant increase in *MMP2* and *MMP9* expression in the RSA group, this result may reflect limitations in sample type rather than a true absence of biological difference. Goto et al. suggested that protein expression of these *MMPs* in decidua and villi—not gene polymorphisms—may be more indicative of their role in RSA, influenced by inflammatory and microenvironmental signals. ^21^ These observations emphasize the importance of post-transcriptional and local regulatory factors.

Several limitations may explain the lack of statistically significant findings. The peripheral blood samples may not capture local gene expression changes in the uterine environment. Additionally, RSA is a heterogeneous condition with diverse underlying causes. Our sample likely included patients with varying etiologies, which may have masked specific gene-related differences. Future studies should include larger, stratified cohorts and analyze both gene and protein expression in relevant reproductive tissues, as well as epigenetic modifications.

## Conclusion

This study found no significant differences in the peripheral blood expression of *DNMT1*, *DNMT3A, MTHFR, MMP2*, and *MMP9* between women with RSA and healthy controls. These findings suggest that peripheral blood may not be an ideal surrogate for evaluating gene expression changes related to pregnancy maintenance. Although these genes have been implicated in key processes such as DNA methylation and extracellular matrix remodeling, their roles may be tissue-specific and influenced by factors beyond mRNA expression levels. Therefore, future studies should focus on analyzing gene and protein expression in placental and decidual tissues, exploring epigenetic modifications, and considering environmental and immunological contexts. A comprehensive, tissue-based, and multidisciplinary approach is essential to unravel the complex pathogenesis of RSA.

## Ethics statement

The study protocol was approved by the Research Ethics Committees of School of Public Health, Shahid Sadoughi University of Medical Sciences, Yazd, Iran with ethical ID: IR.SSU.SPH.REC.1401.045. All of the participants written (signed) an informed consent before entering the study. ( **See supplementary ethic certificate**).

The ethical principles of the Declaration of Helsinki were applied with respect to the confidentiality and veracity of the data collected during the course of the study, which are faithfully presented. Personal identity data and patient privacy were protected. Authorship contributions and transparency in conflicts of interest were reported. ^15^

## Data Availability

The underlying data for this study are available via Figshare: GraphPad Prism results for “Peripheral Blood Expression of MMPs and DNA Methylation-Related Genes in Women with Recurrent Spontaneous Abortion: A Case-Control Study” DOI: doi.org/10.6084/m9.figshare.29262128 ^22^ The project contains the following underlying data:
• Processed Ct values used for statistical analysis in GraphPad Prism • Group-wise comparisons of gene expression between case and control samples Data are available under the terms of the Creative Commons Attribution 4.0 International license (CC-BY 4.0).
