# Supplementary material for: Peripheral Blood Expression of MMPs and DNA Methylation-Related Genes in Women with Recurrent Spontaneous Abortion: A Case-Control Study
Source: F1000Res. 2025 Oct 6;14:763. Originally published 2025 Aug 4. [Version 2] doi: 10.12688/f1000research.166306.2 (PMC12521897; doi:10.12688/f1000research.166306.2)
Supplement: Peer review history

# Reviewer report: Reviewer response for version 2

**DOI:** 10.5256/f1000research.189089.r420793

**Published:** 2025-10-14

**Authors:** Nafisa Huseni Balasinor

**Affiliations:** 1 ICMR-National Institute for Research in Reproductive & Child Health, Mumbai, Maharashtra, India

The authors have responded to the comments and revised the manuscript. The manuscript may be accepted for indexing

Is the work clearly and accurately presented and does it cite the current literature?

Partly

If applicable, is the statistical analysis and its interpretation appropriate?

Yes

Are all the source data underlying the results available to ensure full reproducibility?

No source data required

Is the study design appropriate and is the work technically sound?

Partly

Are the conclusions drawn adequately supported by the results?

Partly

Are sufficient details of methods and analysis provided to allow replication by others?

Yes

Reviewer Expertise:

Reproductive endocrinology, epigenetic and paternal factors in pregnancy outcome, like recurrent spontaneous abortions and preeclampsia

I confirm that I have read this submission and believe that I have an appropriate level of expertise to confirm that it is of an acceptable scientific standard.

# Reviewer report: Reviewer response for version 1

**DOI:** 10.5256/f1000research.183273.r403505

**Published:** 2025-09-03

**Authors:** Nafisa Huseni Balasinor

**Affiliations:** 1 ICMR-National Institute for Research in Reproductive & Child Health, Mumbai, Maharashtra, India

The manuscript has investigated the expression of 5 genes in peripheral blood  of women experiencing Recurrent spontaneous abortions ((RSA) and compared with non pregnant proven fertile women without any history of RSA and they did not find any significant change in the expression of the genes studied

 The MS is well carried out and well written, however the following aspect which needs to be addressed

 1. The authors have studied gene expression in pheripheral blood and that too 3 months after miscarriage, which is too long an interval to study gene expression. The authors have acknowledged that studying in peripheral blood is a limitation of their study and not an ideal tissue to study gene expression. However, the 3 months time interval after miscarriage may not be appropriate

 Other points to be addressed

 1. Introduction: 2nd paragraph last sentence. The statistics is from one study and quite old after which many studies have been published on DNA methylation and RSA/RPL  so cannot be taken as globally. Need to cite recent study on DNA methylation in the context of RSA

 2. GAPDH used as internal reference genes: Was it ensured that expression of this gene is not affected in RSA cases. Usually more than one reference gene is used

 3. Discussion: 2nd Paragraph: "...showed a higher expression of DNMT1 and DNMT3A in the RSA group, the differences were not statistically significant. This aligns with findings from Li et al., who reported global hypomethylation and reduced DNMT expression in chorionic villi and decidua of URPL patients, along with increased TET enzyme activity. Additionally, inhibition of DNMT1 in

 early pregnancy models has been shown to reduce DNA methylation and impair embryonic attachment, underscoring the critical role of balanced methylation".

 Comment: In the present study a non significant increase in DNMTs would lead to hypermethylation which does not align with the cited 2 studies

 4. Discussion: MTHFR expression: The authors conclude that their finding highlight potential disconnect between MTHFR promoter methylation and expression. However, they should be cautious as they have not analyzed methylation and the time interval of 3 months in the present study maybe the reason for observing no change in expression.

Is the work clearly and accurately presented and does it cite the current literature?

Partly

If applicable, is the statistical analysis and its interpretation appropriate?

Yes

Are all the source data underlying the results available to ensure full reproducibility?

No source data required

Is the study design appropriate and is the work technically sound?

Partly

Are the conclusions drawn adequately supported by the results?

Partly

Are sufficient details of methods and analysis provided to allow replication by others?

Yes

Reviewer Expertise:

Reproductive endocrinology, epigenetic and paternal factors in pregnancy outcome, like recurrent spontaneous abortions and preeclampsia

I confirm that I have read this submission and believe that I have an appropriate level of expertise to confirm that it is of an acceptable scientific standard, however I have significant reservations, as outlined above.

# Response

**Published:** 2025-09-14

**Authors:** Farzaneh Fesahat

**Affiliations:** Shahid Sadoughi University of Medical Sciences, Yazd, Iran, Iran

We sincerely appreciate the reviewer’s insightful comment regarding the timing of sample collection. As acknowledged in the manuscript, one of the limitations of this study is the use of peripheral blood as the tissue source for gene expression analysis, which may not fully reflect molecular changes occurring in reproductive tissues such as the decidua or placenta. However, our primary objective was to investigate persistent or baseline alterations in the expression of genes related to DNA methylation and extracellular matrix remodeling that may predispose individuals to recurrent spontaneous abortion (RSA), rather than transient changes associated with the acute phase of miscarriage.

 The three-month interval between the last miscarriage and sample collection was intentionally chosen to minimize the confounding effects of acute inflammation, hormonal fluctuations, and psychological stress, which are known to influence gene expression. Previous studies have suggested that certain epigenetic modifications and gene expression profiles can remain altered for extended periods following reproductive events. Therefore, we aimed to capture potentially stable molecular signatures that may persist beyond the acute phase, while avoiding short-term physiological noise. We have clarified this rationale in the revised Discussion section.

 1. We appreciate the reviewer’s valuable observation regarding the need to reference more recent studies on DNA methylation in the context of recurrent spontaneous abortion (RSA).  we have revised the final sentence of the second paragraph in the Introduction to include recent findings.

 Specifically, we have added the following two references to support the current understanding of epigenetic dysregulation in RSA:

 -         Qin M, Chen W, Hua L, Meng Y, Wang J, Li H, et al. DNA methylation abnormalities induced by advanced maternal age in villi prime a high-risk state for spontaneous abortion. 2023;15(1):44.

 -          Zhou Q, Xiong Y, Qu B, Bao A, Zhang YJFii. DNA methylation and recurrent pregnancy loss: a mysterious compass? 2021;12:738962.

 2. We thank the reviewer for raising this important point regarding the selection of GAPDH as the internal reference gene. During the initial setup phase, we randomly selected a subset of samples and evaluated the gene expression levels of both *GAPDH* and *ACTB *as candidate reference genes. The results showed no variation in the expression levels of either *GAPDH* or *ACTB *between RSA and control samples. Due to limitations in available cDNA quantity and Based on this preliminary assessment and the widespread use of *GAPDH* in studies ( https://doi.org/10.1186/s12864-021-07801-0), ( https://doi.org/10.5281/zenodo.13904990 ) with similar sampling, GAPDH was finally selected for final normalization in this study.

 3. We have clarified this point in the revised Discussion (paragraph 2) and appreciate the reviewer’s insight in helping us refine the interpretation of our findings.

 4. We thank the reviewer for this important and thoughtful comment. We fully acknowledge that our study did not include direct analysis of MTHFR promoter methylation, and therefore any inference regarding a disconnect between methylation and gene expression must be interpreted with caution. Our statement in the Discussion was intended to highlight a hypothesis based on previous literature, including studies that have reported altered methylation of the MTHFR promoter region in RSA patients. However, we agree that without direct methylation data, such conclusions remain speculative.

 Additionally, the reviewer’s point regarding the three-month interval between miscarriage and sample collection is well taken. It is possible that transient changes in MTHFR expression, which may occur during or shortly after miscarriage, were no longer detectable at the time of sampling. Our study aimed to capture more stable or baseline expression patterns, but we recognize that this timing may have limited our ability to observe dynamic regulatory changes.

 We have revised the Discussion section to clarify these limitations and to avoid overinterpretation of the observed expression data. We appreciate the reviewer’s insight in helping us refine the interpretation of our findings.

# Reviewer report: Reviewer response for version 1

**DOI:** 10.5256/f1000research.183273.r403503

**Published:** 2025-09-02

**Authors:** Noor Nihad Baqer

**Affiliations:** 1 Research and Technology Center of Environment, Baghdad, Iraq

Dears This research represent scientific article and its elegance in writing but there is some notes ,the author can added other referances in introduction speciality of the region near from iran such as iraq due to similar in race these referances such as : 

 1-HLA-G rs1063320 Polymorphism with the incidence of Recurrent Spontaneous Abortion (RSA) in Iraqi Women

 2-Association between Methylenetetrahydrofolate Reductase (MTHFR) and 5-Methyltetrahydrofolate-Homocysteine Methyltransferase Reductase (MTRR) Polymorphisms in Iraqi Patients with COVID-19

 3-The association between serum level and genetic variation of IL-3 (rs40401) in recurrent abortion women infected with toxoplasmosis in Iraq

 4- Association between genetic polymorphism of IL-27 (rs153109) and toxoplasmosis in Iraqi women with recurrent abortion

Is the work clearly and accurately presented and does it cite the current literature?

Yes

If applicable, is the statistical analysis and its interpretation appropriate?

Yes

Are all the source data underlying the results available to ensure full reproducibility?

Yes

Is the study design appropriate and is the work technically sound?

Yes

Are the conclusions drawn adequately supported by the results?

Yes

Are sufficient details of methods and analysis provided to allow replication by others?

Yes

Reviewer Expertise:

immunogenetics

I confirm that I have read this submission and believe that I have an appropriate level of expertise to confirm that it is of an acceptable scientific standard.

# Response

**Published:** 2025-09-14

**Authors:** Farzaneh Fesahat

**Affiliations:** Shahid Sadoughi University of Medical Sciences, Yazd, Iran, Iran

We sincerely thank the reviewer for their thoughtful and constructive feedback. We fully agree with the importance of incorporating regional studies, particularly from neighboring countries such as Iraq, given the shared genetic and ethnic backgrounds that may influence gene polymorphism patterns. In line with this valuable suggestion, we have cited the recommended article—"Association between Methylenetetrahydrofolate Reductase (MTHFR) and 5-Methyltetrahydrofolate-Homocysteine Methyltransferase Reductase (MTRR) Polymorphisms in Iraqi Patients with COVID-19"—in the revised manuscript.
